# Thoracic transforaminal epidural steroid injection for management of thoracic spine pain: A multicenter cross-sectional study of short-term outcomes

**DOI:** 10.1016/j.inpm.2021.100004

**Published:** 2021-12-16

**Authors:** Josh Levin, John Chan, Lisa Huynh, Matt Smuck, Jayme Koltsov, Bilge Kesikburun, Graham E. Wagner, Marc Caragea, Keith Kuo, Zachary L. McCormick, Byron Schneider, Evan Berlin, D.J. Kennedy, Serdar Kesikburun

**Affiliations:** a Department of Orthopaedic Surgery, Stanford University, 450 Broadway St., Pavilion C; 4th Floor, MC 6342, Redwood City, CA, 94063, USA; b Department of Neurosurgery, Stanford University, USA; c Ankara City Hospital, Universiteler Mahallesi 1604. Cadde No: 9, Cankaya, Ankara, 06800, Turkey; d Division of Physical Medicine and Rehabilitation, University of Utah, 30 N 1900 E, Salt Lake City, UT, 84132, USA; e Department of Physical Medicine and Rehabilitation, Vanderbilt University, 2201 Children's Way, Suite 1318, Nashville, TN, 37212, USA; f University of Health Sciences Turkey, Gaziler Physical Therapy and Rehabilitation Training and Research Hospital, Universiteler Mahallesi, Lodumlu Yolu, 29 Ekim Sokak No:1, Cankay, Ankara, 06800, Turkey

**Keywords:** Thoracic, Transforaminal, Epidural, Steroid, Injection

## Abstract

**Background:**

Thoracic transforaminal epidural steroid injections (TFESIs) are procedures performed for the treatment of thoracic spine pain (TSP). The literature on these interventions is sparse.

**Purpose:**

To report outcomes of thoracic TFESIs for TSP indications.

**Study design:**

Multicenter, retrospective, cross-sectional cohort study.

**Patient sample:**

Consecutive patients receiving thoracic TFESIs at three academic spine centers.

**Outcome measures:**

The primary outcome was the proportion of patients reporting at least 50% improvement in NRS pain score at short-term follow-up (>1 week, <3 months post-injection).

**Methods:**

A chart review was performed of consecutive patients who underwent a thoracic TFESI over a 4- to 10-year time period at three academic spine centers and had reported an NRS pain score at short-term follow-up.

**Results:**

Overall, 19/64 patients (30% [95% CI 20–42%]) experienced ≥50% relief following the injection at a median 22 days follow-up. 42% [95% CI 31–54%] experienced at least a 2-point improvement in NRS score. There was a slight improvement in median NRS scores from pre-to post-procedure of −1 (IQR -3, 0), from 6/10 to 5/10 (p ​< ​0.001). The success rate (≥50% pain relief) was 36% [95%CI 22–52%] in those with a disc herniation as compared to 21% [95%CI 10–40%]) in those with degenerative stenosis; however, the difference did not reach statistical significance. There was a trend towards a greater success rate in those who were employed vs. unemployed (43% [95% CI 27–61%] vs. 19% [95% CI 9–36%]).

**Conclusions:**

This is the largest series reporting outcomes from thoracic TFESI to date. Overall, the observed success rate was low compared to known success rates associated with TFESI for the treatment of pain at cervical and lumbar spinal regions.

## 1 Introduction

Although not nearly as frequent as low back pain or neck pain, thoracic spine pain (TSP) does affect a substantial number of individuals, with a lifetime prevalence ranging from 15 to 20% [1]. TSP is also a major occupational health issue affecting 1 in 5 women and 1 in 10 men in the working population [2].

Thoracic radicular pain most commonly originates from a disc herniation, degenerative stenosis, or vertebral compression fracture [3]. The presentation of radicular pain in the thoracic region is more common with lateral disc herniations, often associated with some amount of axial pain, and the majority of thoracic disc herniations are below the T8 level [4]. Signs and symptoms of myelopathy such as ataxia, bowel or bladder symptoms, and sensory or motor dysfunction in the lower limbs are uncommon [5].

Both conservative and surgical treatment modalities may be used to manage TSP caused by disc herniations or degenerative stenosis. The most utilized conservative interventions are medications, postural optimization, and exercise. In more severe or refractory cases, epidural steroid injections may be used. Fluoroscopically guided transforaminal epidural steroid injection (TFESI) is a target-specific treatment for radicular pain in which steroid is placed along the spinal nerve root. There is strong evidence that TFESI is an effective treatment for patients with lumbar radicular pain [6,7], and lower quality evidence of possible benefit for cervical radicular pain [[8], [9], [10], [11]]. However, there is a paucity of literature on thoracic TFESIs for the treatment of TSP. The minimal existing literature addresses the use of thoracic TFESI for the treatment of post-herpetic neuralgia [12,13], but not TSP caused by disc herniations or degenerative stenosis. As such, the present study aimed to report the effectiveness of fluoroscopically guided thoracic TFESIs in patients with TSP caused by one of these two conditions.

## 2 Methods

This study was approved by the institutional review boards at three academic medical centers (IRB number 48537 ​at Stanford University, IRB number 00069703 ​at University of Utah, and IRB number 212263 ​at Vanderbilt University). The study was conducted according to the Declaration of Helsinki. A retrospective cross-sectional electronic chart review was performed on consecutive patients who underwent a thoracic TFESI between January 2010 and October 2018 ​at Stanford University, between January 2010 and May 2020 ​at University of Utah, and between October 2016 and June 2020 ​at Vanderbilt University. Inclusion criteria were: aged 18 years old or older, underwent a thoracic TFESI, had cross-sectional imaging within 12 months of receiving their thoracic TFESI, and had follow-up data within 3 months of the injection. Due to the vague nature of thoracic radicular pain, and the limitations of obtaining the data retrospectively via chart review, a clear definition of thoracic radicular pain with distal radiating symptoms in a nerve root distribution was not set as an inclusion criterion. Exclusion criteria were: missing numeric rating scale (NRS) pain scores before or after the thoracic TFESI, and diagnostic injection without steroid. There was not a minimum duration follow-up time as an exclusion criterion, however no patient had follow-up data collected prior to 7 days post-injection.

All procedures were performed by fellowship-trained spine physiatrists with extensive experience in spinal interventions at three large academic physical medicine and rehabilitation spine clinics. The intervertebral level and side of the injection were determined by the treating physician based on clinical evaluation and imaging findings. All injections were performed at the level of the primary imaging abnormality, or at the level below. The injections were performed according to Spine Intervention Society Guidelines [14]. In short, patients were placed in the prone position. Oblique fluoroscopic views were obtained to view the targeted foramen, and the needle was guided into place below the pedicle. Contrast was injected through small bore extension tubing under continuous fluoroscopic imaging in order to confirm epidural and extravascular needle placement. Digital subtraction imaging and anesthetic test doses were used at the discretion of the treating physicians. Following confirmation of goal needle tip position, the steroid was injected. Dexamethasone was used for all injections other than 2 injections that were performed prior to 2014.

MRI or CT images were reviewed independently by two fellowship trained spine physiatrists. A primary diagnosis related to the relevant spinal pathology was defined as “disc herniation,” or “degenerative stenosis”. A third fellowship trained spine physiatrist was used as a tiebreaker for disagreements. The imaging was reviewed at the level of the thoracic spine that was targeted during the injection.

The severity of pain was evaluated using a numeric rating scale (NRS) ranging from 0 to 10, before the procedure and within 3 months afterwards at a follow-up visit. The pre-procedure NRS pain score was obtained from the patient's most recent clinic documentation, or from the pre-procedure history and physical documentation. The primary outcome measure was pain relief as defined by ​≥ ​50% improvement in NRS pain score at follow-up. Secondary outcome measures were pain relief as defined by ​≥ ​80% improvement in the NRS pain score, and pain relief as defined by ​≥ ​2-point improvement in the NRS pain score.

In addition to pre- and post-injection pain scores and MRI findings, we also collected the following information in our chart review: age, BMI (body mass index), gender, smoking status, and work status.

### 2.1 Statistical analysis

All analyses were conducted in SAS version 9.4 (Cary, NC, USA) with a two-sided level of significance of 0.05. A simple kappa statistic and 95% confidence interval was calculated to measure inter-rater reliability for the MRI analysis of a primary diagnosis of “disc herniation” vs “degenerative stenosis”. Agreement was defined as almost perfect for kappa above 0.90, strong for 0.80–0.90, moderate for 0.60–0.79, weak for 0.40–0.59, minimal for 0.21–0.39, and none for 0–0.20 [15].

The proportions of patients achieving ≥50% and ≥80% improvement in pain as measured by the NRS values were calculated with Wilson score 95% confidence intervals (95% CI), as was the proportion of patients achieving the minimal clinically important change (MCIC) of at least a 2 point reduction in NRS pain score [16]. Univariate relationships with ≥50% or ≥80% improvement were assessed with chis-squared and Fisher's exact tests for categorical variables and independent samples t-tests for continuous variables (or Mann-Whitney U tests if non-normally distributed). For relationships with the raw change in NRS pain, relationships with categorical variables were assessed with Mann Whitney U and Kruskal Wallis tests, and relationships with continuous variables were assessed with linear regression.

## 3 Results

A total of 3,545 patients were identified through our search for CPT code 64479 (transforaminal epidural injection, cervical or thoracic). Of these, 88 underwent a thoracic transforaminal epidural injection (the remainder underwent cervical transforaminal epidural injections). Six patients were not included in the analysis due to follow-up data obtained beyond the 90-day post-injection inclusion criterion. Of the remaining 82 patients, a total of 18 patients were excluded for the following reasons: no available follow-up data (n=7), cross-sectional imaging no longer available to review (n=3), pathology other than disc herniation or degenerative stenosis (n=3), diagnostic injection without steroid (n=3), and MRI prior to 1 year before the injection (n=2). 64 patients remained and were included in the analysis. See Table 1 for a summary of demographic and clinical data. A primary diagnosis of herniated disc was slightly more common than a primary diagnosis of degenerative stenosis (Table 1). The grading of thoracic spinal imaging pathology as herniated disc vs. degenerative stenosis showed moderate inter-rater reliability with a kappa statistic of 0.78 [95% CI 0.63–0.93]. Injections were performed at all levels of the thoracic spine other than T2-3 (Table 2.) Injections were performed at only one spinal level on all but 6 patients for whom 2 levels were injected. Thirty-eight injections were performed unilaterally, and 26 were performed bilaterally.

**Table 1:** Baseline demographic and clinical information, presented as n (%) for categorical variables, and means and 95% CIs for continuous variables (or medians and interquartile ranges (IQR) where noted).

Age (years)	58 [54, 62]
BMI	26.7 [25.3, 28.2]
Female	34 (53%)
Current smoker	5 (8%)
Currently employed	28 (48%)
Primary Diagnosis: Degenerative Stenosis Disc Herniation	28 (44%) 36 (56%)
Baseline NRS pain score [median (IQR)]	6.0 (5, 8)
Duration of pain in months [median (IQR)]	12 (6, 36)

BMI ​= ​body mass index; NRS ​= ​numeric rating scale.

**Table 2:** Number of injections performed at each level, presented as n (%) (6 patients had 2 levels injected; total number of injections ​= ​70).

Level of Injection	Number of Injections
T1-2	1 (1%)
T2-3	0 (0%)
T3-4	3 (4%)
T4-5	2 (3%)
T5-6	6 (9%)
T6-7	7 (10%)
T7-8	5 (7%)
T8-9	10 (14%)
T9-10	6 (9%)
T10-11	5 (7%)
T11-12	11 (16%)
T12-L1	14 (20%)

Of the 64 patients, 30% [95%CI 20–42%] had a successful outcome as defined by ​≥ ​50% relief in pain, and 11% [5–21%] had ≥80% relief in pain at short-term follow-up. Forty-two percent [95% CI 31–54%] experienced at least a 2-point improvement in NRS pain score. When examining median change in NRS pain score, there was a statistically significant reduction from pre-to post-procedure of −1 (IQR -3, 0), from 6/10 to 5/10 (p ​< ​0.001), however this did not represent a clinically significant improvement (Table 3).

**Table 3:** Frequency and percentages of patients meeting improvement thresholds (50%, 80%, MCIC) in NRS pain. Percentages are shown with Wilson score 95% confidence intervals. Descriptive statistics are also presented for the raw NRS pain scores and follow up time.

Achieved ≥50% NRS pain improvement	n ​= ​19 30% [95% CI 20–42%]
Achieved ≥80% NRS pain improvement	n ​= ​7 11% [95% CI 5–21%]
Achieved ≥2 point NRS pain improvement	n ​= ​27 42% [95% CI 31–54%]
Baseline NRS pain [median (IQR)]	6 (5, 8)
Follow-up NRS pain [median (IQR)]	5 (3, 7)
Follow-up time in days [median (range)]	22 (7, 75)

NRS ​= ​numeric rating scale; 95% CI ​= ​95% confidence interval; IQR ​= ​interquartile range.

36% [95% CI 22–52%] of patients with a primary diagnosis of disc herniation achieved ≥50% pain relief, versus 21% [95% CI 10–40%] of patients with a primary diagnosis of degenerative stenosis; however, this result did not reach statistical significance (p ​= ​0.20). There was a trend towards superior short-term pain reduction in those who were employed (43% [95% CI 27–61%] vs 19% [95% CI 9–36%] with ≥50% pain relief, p ​= ​0.09). When considering the median improvement in pain score, the relationship with employment reached significance (−2.0 employed versus 0.0 unemployed; p ​= ​0.035).

A subgroup analysis was performed to evaluate for differences in the primary outcome (≥50% improvement in NRS pain score) in patients whose follow-up was obtained less than 2 weeks after the injection, between 2- and 4-weeks post-injection, between 4- and 8-weeks post-injection, and between 8- and 12-weeks post-injection. No significant differences were seen (Table 4).

**Table 4:** Percentages of patients who achieved a successful outcome (≥50% NRS pain improvement) based on different follow-up time periods. Percentages are shown with Wilson score 95% confidence intervals.

Follow-up less than 2 weeks post-injection	3/11 ​= ​27% [95% CI 1–53%]
Follow-up 2–4 weeks post-injection	8/31 ​= ​26% [95% CI 11–41%]
Follow-up 4–8 weeks post-injection	7/18 ​= ​39% [95% CI 16–62%]
Follow-up 8–12 weeks post-injection	1/4 ​= ​25% [95% CI 0–67%]

NRS ​= ​numeric rating scale; 95% CI ​= ​95% confidence interval.

Twenty-one out of 64 patients had a repeat injection, and 13 of these were performed within 2 months of the first injection. Follow-up data after the repeat injection was not available for six of these patients. Of the 15 patients with follow-up data after the repeat injection, six had ≥50% pain relief (1 had ≥80% pain relief).

One patient experienced asymptomatic bradycardia that resolved without intervention, consistent with a vasovagal reaction. No other complications were reported.

## 4 Discussion

The available literature on outcomes from thoracic TFESIs is sparse. Wang et al. reported on complications from 296 thoracic TFESIs. While they did report “partial or complete pain relief” in 88% of patients (62% with partial relief, 26% with complete relief), the assessment was performed immediately after the procedure, and neither short nor long-term outcome measurements were collected [17]. Other previous reports include studies on procedural technique [18], radiographic outcomes [13,19], or case reports [12,[20], [21], [22]]. Our study represents the largest reported cohort to date on short-term pain outcomes following thoracic TFESI, and the multicenter design is a strong feature of our study. Notably, only 64 patients with pre- and post-injection NRS pain scores were identified in a 4- to 10-year time period at three busy academic PM&R spine centers; this is indicative of the low incidence of presumed thoracic radicular pain refractory to non-invasive treatment strategies.

Our overall success rate of 30% is low compared to the approximately 50–60% success rates that have been reported in the outcome literature when TFESI is performed for radicular pain in other spinal regions [[6], [7], [8], [9],^11^]. Given the complexity of TSP, these results are not unexpected. Thoracic pain is often vague, and identifying a specific level of pathology is challenging. These factors may have contributed to the lower success rates.

The only variable that was significantly associated with better outcomes was employment status, with better results in patients who were employed in median pain score improvement, but not in the categorical primary outcome. Unfortunately, our chart review was not able to determine the specific reasons for unemployment status for most patients. Patients with a primary diagnosis of disc herniation were more likely to report ≥50% pain relief than patients with a primary diagnosis of degenerative stenosis, but this finding was not statistically significant with the current sample size. Future studies with larger sample sizes could aid in patient selection for thoracic TFESIs.

Our study has limitations. First, the clinical evaluation of thoracic spine pain is challenging, as symptoms are often ill-defined, especially when compared to cervical and lumbar radicular pain. Therefore, the definition of TSP is complex, and in our retrospective analysis, it was not predefined.

Additionally, the retrospective nature of our analysis contains the possibility of bias. However, all consecutive patients who met inclusion criteria were analyzed, and the data was collected prospectively, thereby reducing the possibility of such bias. Also, our multicenter design is a strength of our study that limits this bias. Because of the cross-sectional nature of our study, the intervention and the outcomes were evaluated simultaneously, and outcomes were measured at varying time points between 7 days and 3 months. NRS pain scores were obtained from the pre-procedure office visit documentation, or from the pre-procedure history and physical examination documentation, and these pain scores may not have accurately reflected the patients’ typical pain scores. We did not have a control group, and some patients who experienced success may have improved due to natural history alone. Similarly, we did not control for co-interventions after the injection, which also could have influenced the observed success rates. Lastly, we assessed short-term outcomes, but did not evaluate outcomes beyond 3 months post-injection.

The sample size of 64 patients provided power to determine the proportion of patients having ≥50% pain relief to within a 95% confidence interval of roughly ±0.1. However, the sample size was underpowered for the univariate tests with categorical baseline covariates. For example, the smallest detectable difference in the proportion of patients having ≥50% pain relief was 0.36 between those with a primary diagnosis of disc herniation versus degenerative stenosis (as compared to the difference of 0.15 observed in the study). We attempted to perform multivariable models to adjust for patient factors significant in the univariate tests, however we were severely underpowered for these models, and they did not provide additional insight.

While our study is the largest reported outcome series on thoracic TFESIs, and it showed a complication rate of only 1.6% [95% CI 0–4.6%] (a self-limited vasovagal reaction), it is not nearly large enough to comment on the safety of the procedure.

## 5 Conclusion

The overall short-term success rate of meaningful pain reduction associated with thoracic TFESIs is low compared to TFESIs at other spinal segmental regions. Future studies with larger sample sizes would be able to better elucidate patient factors associated with TFESI success and confirm whether there may be a trend towards more likely success in patients with a primary diagnosis of disc herniation compared to those with degenerative stenosis.

## Declaration of competing interest

The authors declare that they have no known competing financial interests or personal relationships that could have appeared to influence the work reported in this paper.
